# Territorial Dynamics and Stable Home Range Formation for Central Place Foragers

**DOI:** 10.1371/journal.pone.0034033

**Published:** 2012-03-30

**Authors:** Jonathan R. Potts, Stephen Harris, Luca Giuggioli

**Affiliations:** 1 Bristol Centre for Complexity Sciences, University of Bristol, Bristol, United Kingdom; 2 School of Biological Sciences, University of Bristol, Bristol, United Kingdom; 3 Department of Engineering Mathematics, University of Bristol, Bristol, United Kingdom; University of Utah, United States of America

## Abstract

Uncovering the mechanisms behind territory formation is a fundamental problem in behavioural ecology. The broad nature of the underlying conspecific avoidance processes are well documented across a wide range of taxa. Scent marking in particular is common to a large range of terrestrial mammals and is known to be fundamental for communication. However, despite its importance, exact quantification of the time-scales over which scent cues and messages persist remains elusive. Recent work by the present authors has begun to shed light on this problem by modelling animals as random walkers with scent-mediated interaction processes. Territories emerge as dynamic objects that continually change shape and slowly move without settling to a fixed location. As a consequence, the utilisation distribution of such an animal results in a slowly increasing home range, as shown for urban foxes (*Vulpes vulpes*). For certain other species, however, home ranges reach a stable state. The present work shows that stable home ranges arise when, in addition to scent-mediated conspecific avoidance, each animal moves as a central place forager. That is, the animal's movement has a random aspect but is also biased towards a fixed location, such as a den or nest site. Dynamic territories emerge but the probability distribution of the territory border locations reaches a steady state, causing stable home ranges to emerge from the territorial dynamics. Approximate analytic expressions for the animal's probability density function are derived. A programme is given for using these expressions to quantify both the strength of the animal's movement bias towards the central place and the time-scale over which scent messages persist. Comparisons are made with previous theoretical work modelling central place foragers with conspecific avoidance. Some insights into the mechanisms behind allometric scaling laws of animal space use are also given.

## Introduction

Understanding the mechanisms behind animal territoriality is of great importance to many areas of ecology [1], from conservation biology [2] to epidemiology [3] to predator-prey dynamics [4]. A species is called territorial if each animal, or group of animals, constructs and defends a region of space from conspecific neighbours or possible intruders. Maintaining a territory relies on the animal's ability to exclude conspecifics from the area it occupies. Since the animal needs to spend time moving inside its territory to carry out vital activities such as foraging, continuous monitoring of territory boundaries is not possible. Therefore many animals have evolved mechanisms whereby their territory is identified by visual, auditory or olfactory signals [5], thereby obviating the need for constant border patrolling.

In this paper we focus on a model where the signals are olfactory (scent marks). It is based on an agent-based model of so-called *territorial random walkers*, first introduced in [6], where animals are modelled as lattice random walkers that deposit scent as they move. The scent is only active for a finite amount of time, the so-called *active scent time*, and if a lattice site contains active scent, no other animal may move there. As a result, the terrain naturally subdivides into territories demarcated by the absence of foreign scent. Territories each have a boundary and if two boundaries are juxtaposed, a border is formed. These borders never settle to a stable state. Instead, they continually ebb and flow, albeit at a much slower rate than the animals move. Specifically, the border movement is subdiffusive (i.e. the variance of the border position's probability distribution increases sublinearly with time) since the territories are undergoing an exclusion process [7], [8], whereas the animals move diffusively.

Here, we study a modified version of the territorial random walk model where animals are random walkers with an attraction towards a central place, such as a den or nest site where the animals return occasionally [9], or a core area where animals tend to spend most of their time [10]. Similar to the original territorial random walk model, territories emerge whose borders are continually fluctuating. However with central place attraction, the mean square displacement (MSD), i.e. the variance of the border position's probability distribution, tends towards a finite value, as confirmed by stochastic simulations. This causes stable home range patterns to emerge from the territorial dynamics.

To understand better the precise nature of the emergent home range patterns, we compare stochastic simulations of the many-bodied non-Markovian central place attraction model with an analytic approximation, following [11], [12]. This exploits the time-scale disparity between the rate of animal movement and the slower, subdiffusive territorial borders, to construct an adiabatic approximation for the joint probability distribution of the animal and territory border positions. The model is solved exactly in both 1D and 2D and the resulting marginal distribution for an animal's position allows the *macroscopic* properties of home range size and overlap to be related to the *microscopic* details of the animals' movement and interaction processes. In particular, our analytic expressions can be used to infer the longevity of olfactory messages purely by examining data on animal space use. Furthermore, since various properties of space use are predicted to scale allometrically [13], our theory can also be used to give insights into the mechanisms behind these scaling laws. Our results are compared with previous approaches to modelling conspecific avoidance with reaction-diffusion formalisms [9].

## Results

### Agent-based simulations of territorial central place foragers

Monte Carlo simulations of the territorial random walk system were performed in both 1D and 2D where each animal has a bias of moving towards a central place (CP) (see Methods for details). The MSD of the territory border eventually reached a saturation value that depended on both the strength of attraction towards the CP and the dimensionless quantity 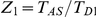 in 1D or 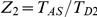 in 2D, where 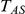 is the active scent time, 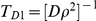 (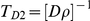) is the diffusive time in 1D (2D) representing the time it takes for an animal to move around its territory, 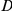 is the animal diffusion constant and 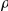 the animal population density. The parameter 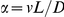 was used to measure the dimensionless strength of CP attraction, where 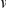 is the drift velocity towards the CP and 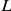 the distance between CPs of two adjacent territories.

**Figure 1:**
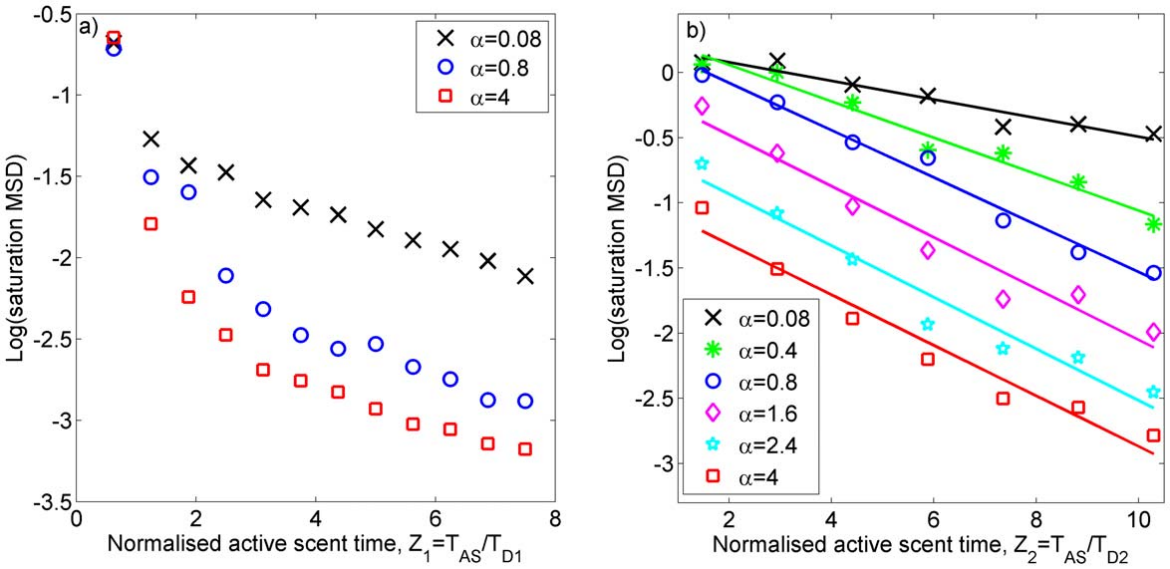
Simulation output for systems of territorial central place foragers. The dependence of the saturation mean square displacement (saturation MSD) 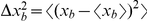 (resp. 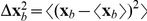) of the dimensionless territory border position 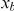 (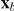) on the dimensionless parameters 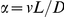 and 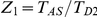 (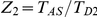) from stochastic simulation output. The notation 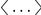 denotes an ensemble average over stochastic simulations. The border movement is non-dimensionalised by dividing by 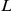, the average distance between central places of adjacent territories. Panel (a) shows output from 1D simulations and panel (b) from 2D simulations. The best-fit lines for the 2D plots are 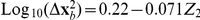 for 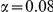, 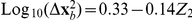 for 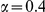, 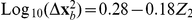 for 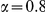, 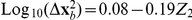 for 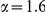, 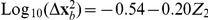 for 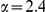, and 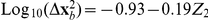 for 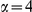.

For a fixed 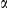, the amount of border movement arises from the ratio of the active scent time to the diffusive time, which is 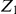 in 1D or 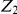 in 2D (figure 1). Increasing 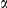 has the effect of reducing the animal's tendency to move into interstitial regions and claim extra territory. This causes the borders to move less on average as each animal keeps to a small core area well within its territory most of the time. Consequently, when plotting the MSD saturation value against 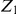 or 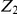, we see that the curves for higher values of 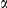 lie below those for lower values (figure 1).

### Dynamics of a central place forager within its territory: a reduced analytic model

By taking into account the fact that the border movement is much slower than that of the animal, we employed an adiabatic approximation to calculate the probability distribution of an animal inside its fluctuating territory borders (see Methods). The simulated animals are identical, so it is sufficient just to model one animal. Since the MSD of each territority border saturates at long times, the animal probability distribution reaches a steady state.

#### Movement in 1D

**Figure 2:**
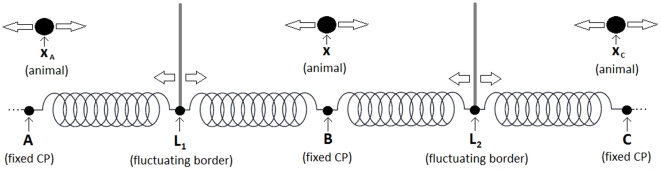
Diagram of the reduced analytic 1D model of territorial dynamics. The CPs are fixed at positions 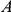, 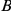 and 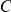 (left to right). The territory borders are intrinsically subdiffusive and have positions 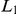 and 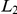. Each animal moves diffusively with a constant drift towards the CP and constrained to move between the two territory borders to its immediate right and left. The position of the animal studied in the main text is denoted by 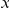. The animals at 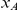 and 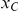 are drawn purely for illustrative purposes. In the Results section, 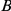 is assumed to be at 0 and 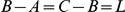.

By fixing the CP at the origin for simplicity, we calculated the steady state 1D dimensionless joint probability density function 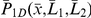 of the left (right) border being at dimensionless positions 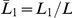 (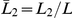) and the animal being at position 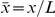 at long times, where 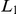, 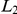 and 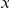 are dimensional parameters and 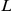 is the distance between CPs of adjacent territories (see figure 2 for an illustration and table 1 for details of notation). This is (see Methods for derivations, here and elsewhere)
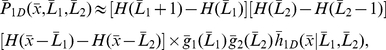
(1)where 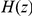 is the Heaviside step function (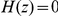 if 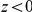, 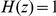 if 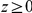), 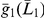 (resp. 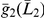) is the probability distribution of the left (right) border and 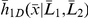 is the probability distribution of an animal being at position 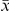, given that the borders are at 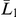 and 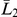. The border probability distributions are given by the following expressions
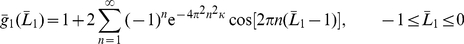
(2)

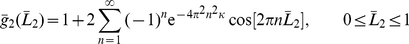
(3)where 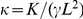, 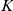 is the border generalised diffusion constant, representing the amount the borders tend to move (see methods), and 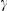 is the rate at which the territory size tends to return to the expected average value. To visualise these distributions, notice that when 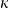 is relatively large, the 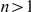 terms are negligible, so that 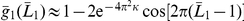 and 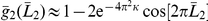.

**Table 1:** Notation glossary.

Symbol	Model	Dimension	Explanation
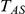	S1,S2	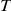	Active scent time: time for which a scent mark is avoided by
			conspecifics.
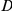	S1,S2,A1,A2	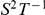	Animal diffusion constant.
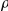	S1,S2	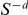	Animal population density in dimension 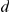 .
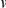	S1,S2,A1,A2	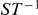	Drift velocity of the animal towards its central place (CP).
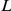	S1,S2,A1,A2	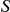	Distance between central places of adjacent territories.
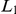 , 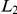	A1	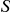	Positions of the left and right borders.
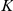	S1,S2,A1,A2	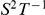	Territory border generalised diffusion constant.
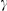	A1,A2	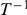	Rate at which territory sizes tend return to the mean size.
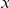	A1	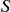	Position of the animal in 1D.
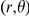	A2	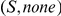	Position of the animal in 2D polar coordinates.
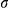	A2	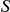	Radius of the territory.
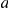	S1,S2	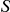	Lattice spacing.
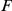	S1,S2	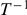	Rate of jumping to the nearest neighbour.
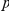	S1,S2	none	Probability of an animal moving towards its CP next jump.
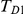	S1	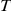	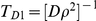 is the 1D diffusive time.
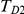	S2	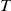	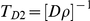 is the 2D diffusive time.
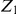	S1	none	Normalised 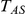 for 1D simulations, 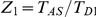 .
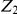	S2	none	Normalised 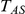 for 2D simulations, 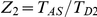 .
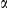	S1,S2,A1,A2	none	Normalised drift velocity 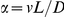 .
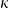	A1,A2	none	Normalised territory border MSD, 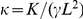 .
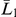 , 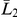	A1	none	Dimensionless positions of the left and right boundaries,
			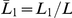 and 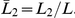
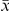	A1	none	Dimensionless position of the animal in 1D, 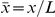 .
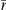	A2	none	Dimensionless radial component of the animal position in
			2D, 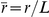 .
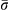	A2	none	Dimensionless radius of the territory, 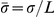 .

Glossary of the various symbols used throught the text. The second column details whether the symbol is used in the 1D simulation model (S1), the 2D simulation model (S2), the 1D analytic model (A1) or the 2D analytic model (A2). The third column gives the dimensions of the parameter, or ‘none’ if it is dimensionless, where 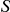 stands for space and 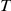 for time.

The probability distribution of an animal being at position 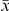, given that the borders are at 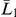 and 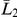, is the following normalised version of a Laplacian distribution with average displacement 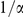


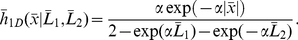
(4)


#### Movement in 2D

In 2D we assumed that the territory is circular, the CP is at the centre of the circle and the border movement is modelled by fluctuations in the territory radius. The steady state dimensionless joint probability density function 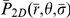 for the territory and the animal at long times is
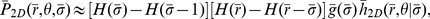
(5)where 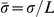 is the dimensionless radius, 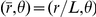 are the dimensionless polar coordinates of the animal, 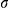 is the radius and 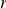 is the radial component of the animal's coordinates. Here,
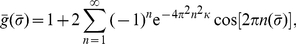
(6)is the probability distribution of the territory radius and
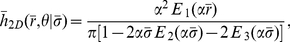
(7)is the probability distribution of the animal being at position 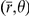 inside a territory of radius 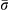, where 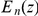 is the special function defined by 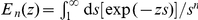. The limit as 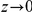 of 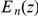 is infinite for 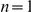 and finite for 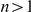. For large 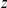, 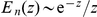 so the limit as 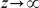 is 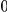 for every 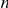.

### The marginal distribution of the animal

Equations (4) and (7) enabled us to calculate the marginal probability distribution of the animal in both 1D and 2D scenarios, where the territory can be anywhere else, by integrating over all possible positions for the territory border. In 1D the dimensionless marginal distribution of the walker at long times is
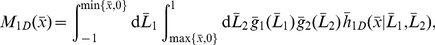
(8)and in 2D, this is
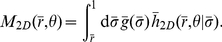
(9)


**Figure 3:**
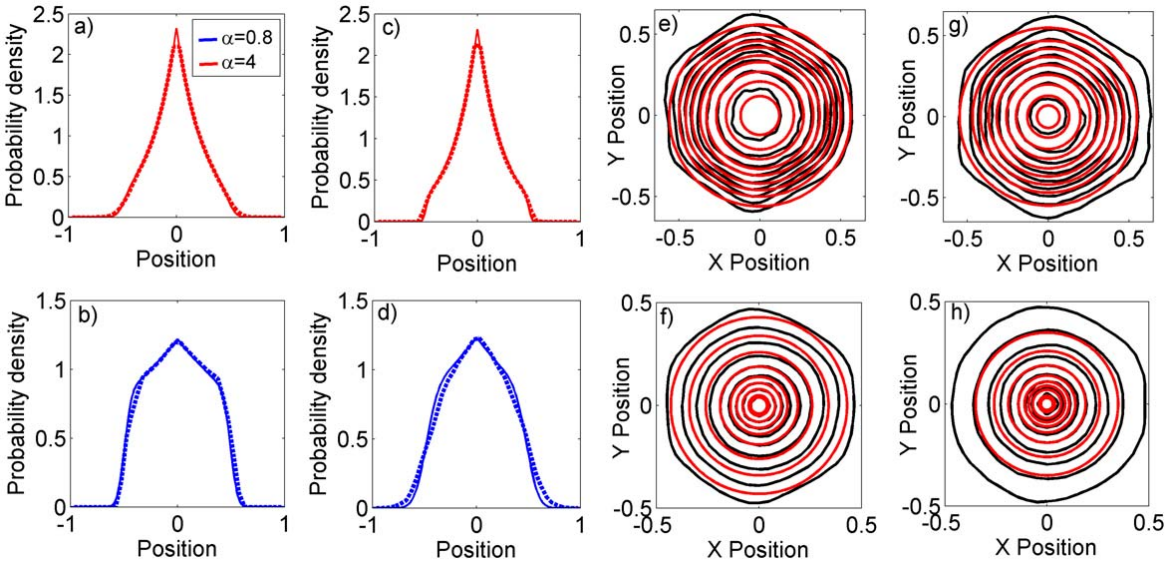
Comparison of the many-bodied simulation system and the reduced analytic model. Saturation marginal probability distributions from simulations of systems of territorial central place foragers are overlaid on the same distributions (equations 8 and 9) from the reduced analytic models. Panels (a–d) compare the two distributions for the 1D system. Dashed lines denote the simulation output and solid lines the analytic approximation. The animal's central place (CP) is at position 0, whereas CPs of conspecifics exist at positions −1 and 1. The distribution decays to 0 at the conspecific CPs, where the animal cannot tread. The values used were (a) 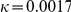, 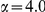, (b) 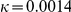, 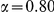 (c) 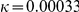, 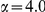 and (d) 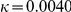, 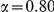. Panels (e–g) compare the two distributions for the 2D system. The black contours show the deciles (i.e. 10%, 20%, 30% etc.) of the height of the probability distribution for the simulation system. The red contours show the same quantities for the analytic approximation. The values used were (e) 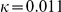, 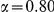, (f) 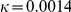, 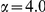, (g) 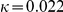, 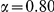 and (h) 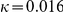, 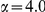. As we increase 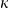 or 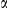, the effect of the adiabatic approximation becomes more apparent, since each red contour is further away from the respective black contour. This is due to the fluctuations of the territory border being more pronounced for higher 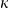 or 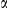.

The effects that the two parameters 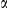 and 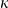 have on the marginal distribution (figure 3) can be characterised by observing that 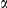 tends to govern the shape of the density function towards the centre of the territory, whereas 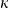 governs the degree to which the distribution tails off sharply (high 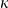) or with a shallow gradient (low 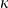).

Expressions (8) and (9) are directly compared with those measured from territorial central place forager simulations. It turns out that the 1D case gives an excellent agreement for all parameter values we tested (figures 3(a–d)). In 2D, a qualitatively close fit is attained only when 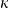 and 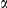 are sufficiently low. For higher 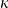 or 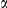 the borders are moving too fast for the adiabatic approximation to be accurate (e.g. figure 3h). However for lower 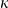 and 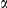, the terrain contains very little interstitial area at any point in time, so the territories are forced to tesselate the plane. Therefore they each form more of a hexagonal than a circular shape (e.g. figure 3e).

### Obtaining active scent time from animal position data

To make use of the present theory, data must be gathered over a sufficiently long period for the animal MSD to saturate. For certain species, the saturation value fails to be reached during the maximal biologically relevant time-window. Male red foxes (*Vulpes vulpes*), for example, may spend parts of the autumn and winter moving outside their territories to cuckold or disperse [14], so territorial dynamics can only be measured reliably from the animal positions during spring and summer when the males tend to stay within their territories. During those two seasons, the tendency to return to the CP is so weak that the animal MSD continues to increase slowly, never settling [6]. In such cases, it is necessary to use methods developed in [6] to analyse the animal territorial system.

However, if the animal MSD does saturate then the marginal distribution (9) can be fitted to the non-dimensionalised distribution of position locations from the data in order to obtain the parameters 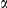 and 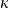. From the theory, the saturation MSD 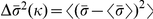 of the territory radius can then be derived from the equation
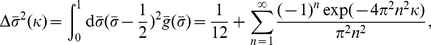
(10)which allows the MSD of the territory radius 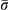 to be computed from 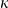. The MSD of 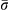 is the analogue, in the analytic model, of the dimensionless territory border MSD 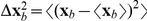 from the simulation model, so we equate 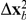 and 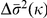. By using the appropriate curve from the simulation output (figure 1b) related to the value of 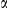 calculated from the data, a value for 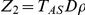 is obtained, from which 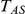 can be derived.

In summary, the active scent time may be obtained from data on animal locations by using the following programme.

1. Fit equation (9) to the data in order to obtain values of 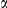 and 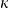.
2. Use this value of 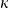 to find the theoretically expected saturation value of the MSD 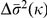 via equation (10).
3. Note that 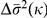 from the analytic model is equal to 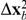 from the simulation model.
4. Identify the best-fit line from figure 1b for the value of 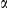 found in step 1.
5. Use this line, together with the value of 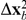 from step 3, to determine the 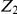-value from figure 1b for the data being studied.
6. Assuming the user also has values for 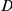 and 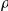 from the data, 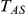 can then be derived from 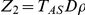.

### Home range patterns and relations to allometry

Since the animal probability distribution reaches a steady state, it is possible to calculate both the size of the resulting home ranges and the degree to which they overlap. By using the 95% MCP method [15], the dimensionless radius of the home range, after dividing by the mean distance between CPs, is given by 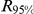, implicitly defined by the following equation
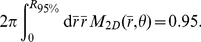
(11)


**Figure 4:**
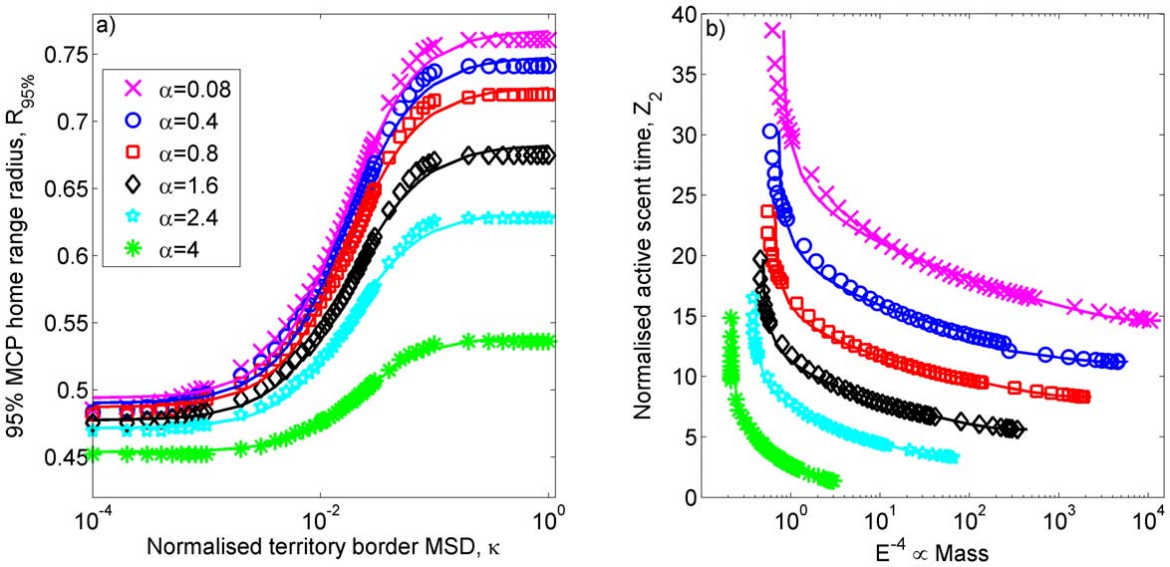
Home ranges and allometry. Panel (a) shows how the radius 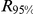 of the normalised (by dividing by the mean distance between CPs) 95% minimum convex polygon home range depends on 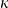 and 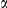 in the 2D analytic model. The various shapes (circles, squares, crosses etc.) show the exact values and the solid lines show the least-squares best-fit sigmoidal curves. Notice that whenever 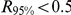, a buffer zone appears between adjacent territories. The proportion of exclusive area 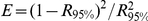 scales with mass [13] so this value is plotted in panel (b) against the dimensionless parameter 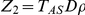 for various 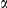. Again, solid lines are derived from the best-fit sigmoidal curves whilst the points denoted by various shapes show exact values.

This allowed us to plot 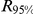 as various functions of 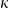, one for each 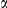 (figure 4a). Each of these can be approximated by a sigmoidal function of 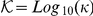. Specifically, 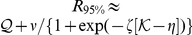, where 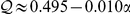, 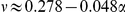, 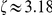 and 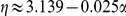 (figure 4a). For certain values of 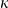 and 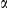, the value of 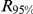 is less than 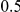, meaning that gaps arise between adjacent territories. These so-called buffer zones have been observed between wolf (*Canis lupus*) territories [4] as a safe place for wolf prey, such as white-tailed deer (*Odocoileus virginianus*), to occupy.

The allometric predictions of [13] show that the fraction of exclusively used area 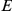 is approximately proportional to 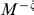 where 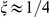 and 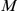 is the mass of a single animal. In our model 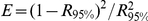 so allometric studies predict 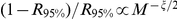. By using the values of 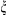 fitted from the large data sets in [13], the value of 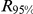 can be estimated for an animal of given mass. Using the trend lines from the simulation plots in figure 1b and equation (10) allows 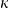 and 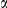 to be related to 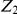, thus estimating how 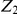 scales with 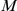, as shown in figure 4b.

In [13] the tendency for larger animals to have a lower proportion of exclusive area in their home ranges was explained intuitively, by noticing that they are less efficient than smaller animals in patrolling their territory to deter conspecifics. That is, the time it takes for a larger animal to get around its territory is greater than that of a smaller animal. In our model, this means the diffusive time, 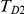, increases with mass. Our results show that this ability to deter conspecifics is also driven by an additional factor: the active scent time. The ability to maintain exclusive area in fact arises from the ratio of 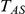 to 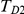. Figure 4b shows that a smaller animal's ability to maintain a higher proportion of exclusive space use arises from maintaining a higher ratio of 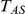 to 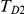, not just a lower diffusive time.

### Comparison with previous approaches

Territoriality in animals with central place attraction has been studied previously in [4] using a reaction-diffusion formalism, which was developed further in [9]. Although both that model and the one presented here use conspecific avoidance mediated by scent marking as the mechanism of territory formation, the present model is built from the individual-level interaction processes, whereas the reaction-diffusion model relies on a mean-field approximation for the scent mark response. Despite the very different natures of their construction and the resulting expressions, we compare the two models by examining the conditions under which they are numerically similar.

In the reaction-diffusion model, 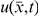 and 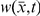 are the dimensionless probability density functions for the left and right animals respectively. In addition, 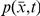 and 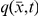 denote the dimensionless densities of the scent of the left and right animals respectively. The dimensionless diffusion constant of each animal is given by 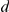 and the dimensionless advection coefficient controlling the strength of motion away from conspecific scent and towards the CP is 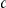. The model also contains a parameter controlling the over-marking response rate: that is, the tendency for animals to scent-mark more having encountered foreign scent. However, since the animals in the model described in the present paper are counter-markers rather than over-markers [16], that is they mark next to conspecific scent but they do not increase marking rate as a response to scent, this parameter is set to 0. With these conditions, the reaction-diffusion system described in [9] has the following dimensionless steady state solution
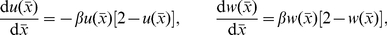
(12)where 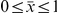, together with the probability conservation conditions
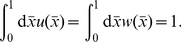
(13)


Equation (12) is equation (6.11) in [9]. The dimensionless parameter 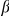 is a function of 5 dimensional parameters, 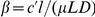, where 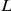 and 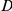 are the same values as used elsewhere in the present study, 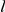 is the scent marking rate for the individual or pack, 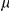 is the rate of scent-mark decay and 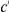 is the strength of attraction towards the CP. The parameter 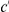 is not the same as the drift velocity 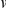 from our model since it has units of 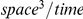 rather than 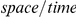. Indeed, the drift velocity at any point 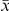 in the reaction-diffusion model is proportional to the strength of foreign scent at 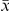 (see equations (4.5) and (4.6) in [9]), whereas in the model studied in the present paper the magnitude of the drift velocity is constant throughout space.

The way the rate of scent deposition is modelled also differs between the two approaches. In the reaction-diffusion model, the rate is independent of the magnitude of the animal's diffusion constant. The biological implication being that as the animal's speed increases, consecutive scent marks will be deposited further apart. In our model, the scent marks are deposited every time the animal has moved a distance 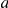 (the lattice spacing), regardless of its speed. The reason for our choice is that it is advantageous for animals to ensure that they deposit territorial messages at regularly spaced intervals so that they leave no gaps in the territory boundaries, which might allow conspecifics to intrude.

Scent decay is also modelled in different ways in the two models. In the reaction-diffusion model the scent decays exponentially, whereas we assume scent is ignored after a fixed period of time (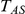). Whilst exponential decay of scent makes sense regarding the decay of the chemicals that produce the odour, a conspecific may ignore a scent mark it can still smell, if the odour suggests that the mark is old and the territory is no longer being defended. For example, such behaviour has been reported for brown hyaenas (*Hyaena brunnea*), whose scent marks may still be detectable by conspecifics over a month later, but who tend to ignore scent that is more than about four days old [17].

Making numerical comparisons of our model with the reaction-diffusion model required a further reduction of our 1D analytic model, since the 1D reaction-diffusion model only represents animal movement in the right-hand (left-hand) half of the left-hand (right-hand) territory. Focussing on the left-hand territory, this required us to simplify our model by fixing 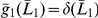 where 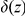 is the Dirac delta function. The resulting marginal distribution for the position of the animal in dimensionless coordinates is
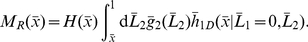
(14)


**Figure 5:**
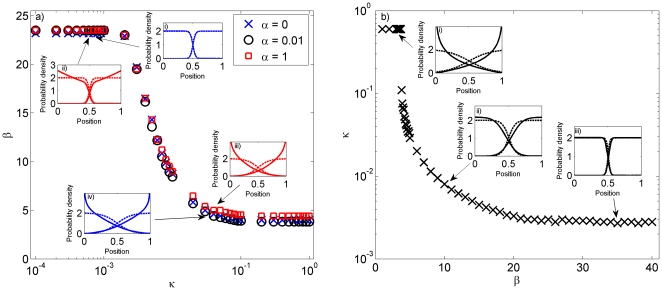
Comparison with a previous model of territory formation. The parameter 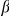 from the reaction-diffusion model introduced in [9] (see also main text) is compared with the parameters 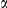 and 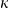 from the 1D analytic model introduced here. Panel (a) shows the 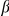-value that gives the best-fit animal marginal distribution curve for each given value of 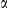 and 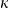. The insets compare the probability distributions for particular values of 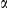 and 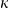, where the solid lines represent our model and the dashed lines the reaction-diffusion model. The values used are (i) 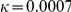, 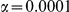, (ii) 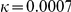, 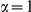, (iii) 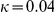, 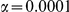, (iv) 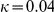, 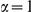. Panel (b) shows the best fit 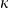-value for a given 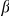. The 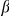-values used for the insets are (i) 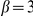, (ii) 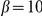, (iii) 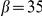. Low values of 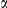 always give a better fit to a given marginal distribution from the reaction-diffusion model than higher values and do not affect the value of 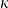 that gives the best fit. Therefore we set 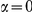 when performing the fitting for panel (b). Low values of 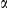 and 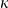 together with high values of 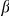 tend to give rise to good fits, but outside this range the two models show quite different results.

This expression is compared with the distribution 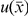 from the reaction-diffusion model, whereas 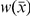 is compared with 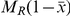. To find the best fit, the square of the difference between the curves of 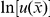 and 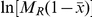 is minimised (figure 5).

Though the two models are qualitatively very different, if 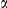 and 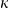 are both very small, it is possible to find a value of 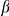 that fits closely (figure 5a). However, if either 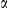 or 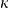 are increased, even the best fit value of 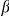 gives a qualitatively different curve. Conversely, for lower values of 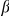, the best fit curve to the model studied here becomes increasingly different to the curve from the reaction-diffusion model (figure 5b).

To explain the similarities in these parameter regimes, the limit case where the scent marks never decay is examined, so that 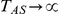 and 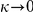. If in addition 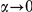, the marginal distribution 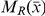 tends towards a step function 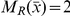 if 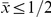 and 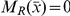 if 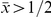. The analogous limit in the reaction-diffusion model is 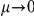 so that 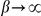. In this limit case, 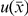 and 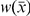 are step functions. By taking the limit numerically as 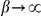, one observes that 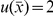 if 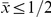 and 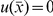 if 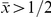 so that 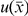 and 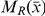 coincide. Similarly, 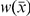 and 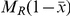 coincide in this limit.

Whilst our model has two parameters, as opposed to one in the reaction-diffusion model, it is possible to collapse our model to one parameter by formally taking the limit 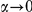 in equation (14), giving the following expression
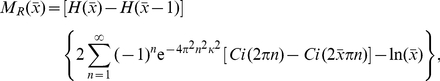
(15)where 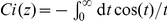 is the cosine integral. This is precisely the limit where the reaction-diffusion model tends to agree best with ours. Plots of equation (15) can be found in the insets (solid lines) of figure (5) for those cases where 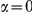.

## Discussion

A central place foraging model with scent-mediated conspecific avoidance has been constructed where the mechanisms of both the animal movement and the interactions are defined at the level of the individual. Territories naturally arise with slowly fluctuating borders whose probability distribution tends towards a steady state. Stable home range patterns emerge, easily enabling us to quantify the home range size and overlap as a function of the underlying individual-level movement and interaction mechanisms. Whilst this is not the first mathematical model of territoriality in central place foragers, nor is it the first where the *movements* are built mechanistically from individual-level processes, [9] it is the first where the conspecific avoidance mechanism is built from *interactions* between individual agents. Though certain predictive inferences have been made using previous approaches, for example regarding what happens when a territory dissolves [18], ours is the first where predictive inferences can be made about the mechanisms of territorial interaction events, in particular the active scent time, from the patterns of animal space-use.

Although deterministic reaction-diffusion equations are in general viable approximations to represent spatio-temporal stochastic processes, they are not well suited to model systems in which the individual components are present in low concentrations (e.g. [19], [20]). This is precisely the situation of decaying scent marks in our model, which are ignored by conspecifics beyond the time 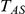. When that happens, the probability density associated with that scent location is identically zero. In other words, in a scent-mediated interaction process the extinction probability for the scent is non-negligible. The system is intrinsically stochastic, and deterministic approximations, where the occupation probability is coupled to a scent mark profile, may not cope with the discrete nature of the interaction events. A reaction-diffusion formalism may thus provide results that are in complete disagreement with the stochastic description (see e.g. [21], [22] in the spatial ecology literature). The particular reaction-diffusion model studied in [9] has been shown here to give very different results to our model, away from the limiting case where scent marks never decay. The similarity in this limit does not come as a surprise, since in this case the scent is never present in low concentrations.

Away from this limit, the choice of model that is most appropriate for a particular data set would depend on both the species involved and the questions to be answered. If one is interested in quantifying both scent marking mechanisms and animal movement processes, a drawback of the reaction-diffusion model is that the dimensionless parameter 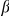 governing the animal space use distribution is a product of 5 (dimensional) parameters, including both the strength of central place attraction and details of the scent marking process. This makes it very difficult, if not impossible, to quantify the scent marking mechanism purely by fitting data to the animal probability density function. On the other hand, the present study gives a clear programme for inferring both the strength of the central place attraction and the active scent time by fitting data on animal space use.

This programme for inferring 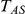 from animal location data was not developed in previous agent-based studies, since the probability distribution of the animal positions never reaches a steady state [6]. In such systems, it is necessary to pick a biologically meaningful time-window over which to measure the extent of home range overlap and thus infer the nature of the border movement and, in turn, the active scent time. This procedure is required for analysing certain animal populations, such as urban red foxes, whose territories, in certain circumstances, may not reach a steady state. However, if a steady state is reached, as shown by a saturating animal MSD, then some aspect of the underlying movement process must be keeping the animal from continually spreading out across the terrain. Such stable home ranges have been reported in a number of species (see e.g. [23]) from wolves (*Canis lupus*) and coyotes (*Canis latrans*) [9] to hispid cotton rats (*Sigmodon hispidus*) [24], cane mice (*Zygodontomys brevicauda*) [26] and Baird's tapirs (*Tapirus bairdii*) [25]. One possible mechanism for ensuring this stability is central place attraction, studied here. It may also be possible that some form of memory mechanism keeps the animal in familiar environments and thus causes the probability distribution to saturate [27], [28].

Our study also gives insights into the mechanisms behind the allometric scaling of exclusive space use. Previous studies had interpreted the observed scaling laws as a consequence of a greater ability for smaller animals to cover their territory regularly, compared to larger animals. However, by quantifying how the scaling arises from the ratio between the active scent time and the territory coverage time, we have shown that the longevity of territorial messages is also a fundamental quantity. Future studies on allometric scaling of space use should also take into account this mechanism of interaction.

## Methods

### The stochastic simulation model

The 1D simulations consisted of 2 animals on a finite lattice with periodic boundary conditions. The central places (CPs) for each animal were uniformly distributed at a distance 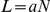 apart, where 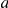 is the lattice spacing and 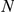 a positive integer. In 2D, 30 animals in a rectangular terrain with periodic boundary conditions were simulated. The CPs were placed at the centroids of a hexagonal lattice, modelling the fact that animal territories tend to be roughly hexagonal in shape [29]. Adjacent CPs were separated by a distance of 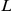. The simulated animals deposited scent at every lattice site they visit, which remained for a time 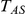, the active scent time. Animals were unable to visit sites that contained scent of another animal. Besides that constraint, at each step an animal moved to an adjacent site at random but its movement was biased towards the CP. In 1D, this meant that there was a probability of 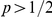 of moving towards the CP and 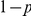 of moving away. In 2D, the movement probabilities were as follows
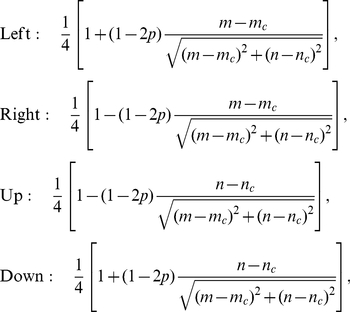
(16)where 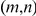 is the position of the animal and 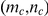 the position of the CP. These probabilities were chosen so that in the continuum limit, they reduce to the form that gives the correct localising tendency in the Holgate-Okubo model (see the section ‘Reduced analytic model in 2D’). In particular, they are independent of the distance the animal is away from the den site. This can be shown by replacing 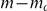 by 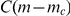 and 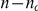 by 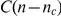 in equations (16), for some non-zero constant 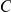, and noticing that all the 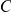-values cancel.

Simulations were run until the MSD of the border had reached a saturation value. Each 1D simulation result was an average of 1,000 simulation runs. In 2D, it was only necessary to average over 100 runs owing to the fact that 15 times as many animals were simulated per run. The simulations were coded in C and compiled on Windows XP OS. To obtain a single saturation MSD value for the 2D simulations took an average of 4 hours 40 minutes CPU time using a 3.0 GHz processor in a 2.96 GB RAM desktop computer.

### The reduced analytical model in 1D

To understand the nature of the animal's movement within its territory borders, we considered a simplified analytic model that uses an adiabatic approximation similar to [11] because the animal moves at a much faster rate than the borders. This meant that the joint probability distribution of the animal and the borders could be decomposed as 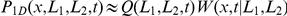 where 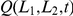 is the probability distribution of the borders to be at positions 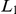 and 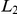 at time 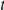, and 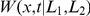 is the probability distribution of an animal to be at position 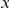 at time 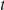 when constrained to move between the borders at 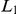 and 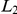.

Following [11], the borders were modelled using a Fokker-Planck formalism, with time-dependent diffusion constant modelling the subdiffusive nature of the border movement, and quadratic potentials modelling the spring forces (figure 2). Since the CP at 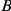 separates 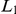 from 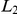, we write 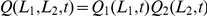 where 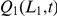 and 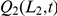 are the probability distributions of 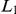 and 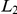 respectively. These are governed by the following equations
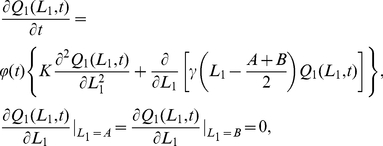
(17)

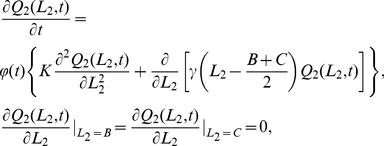
(18)where 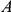 is the position of the CP to the left of 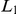, 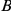 is the position of the CP between 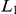 and 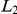, 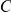 is the position of the CP to the right of 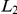, 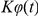 is the time-dependent diffusion constant and 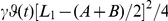 (resp. 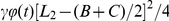) is the quadratic potential for each spring connected to 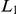 (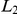). It ensures that the border 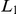 (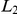) fluctuates around an average position of 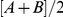 (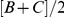). Notice that there are two springs connected to 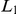 (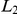), so that the total resulting potential is 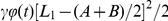 (resp. 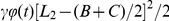). As usual for Fokker-Planck equations (see e.g. [31]), this potential appears in equation (17) (resp. 18) after having been differentiated with respect to 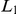 (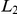), to give 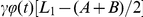 (resp. 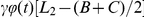). The boundary conditions in equations (17) and (18) ensure that the borders cannot cross over the CPs, since each CP must remain in its territory.


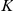
 and 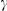 can be measured directly from the simulation model (see e.g. [11]). However, in the steady state solutions (equations 2 and 3), 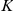 and 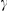 collapse to a single parameter 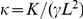. The 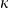 parameter can be derived by first measuring the boundary's saturation MSD from the simulations, and then using equation (10).

Equations (17) and (18) can be solved using the method of characteristics [30]. The general solution to (17) is
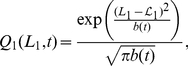
(19)where 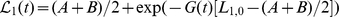, 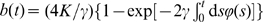, 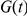 is defined so that 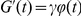 and 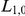 is the initial value for 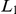 at time 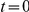. By using the method of images [32] to take account of the boundary condition and assuming, for simplicity, that 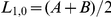, we arrive at the following solution
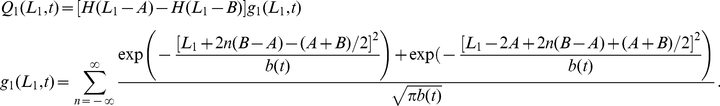
(20)


Similarly,
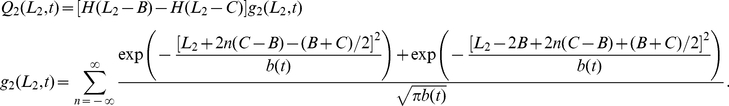
(21)


By making use of the Poisson summation formula [32], equations (20) and (21) can be re-written as follows
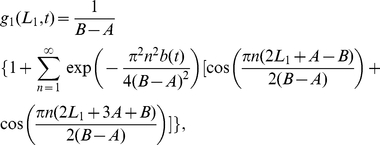
(22)

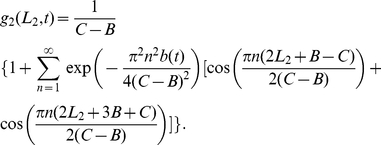
(23)


Since the territories move as tagged objects in a single file diffusion process [6], we have 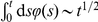 in the 1D system [8]. Therefore the limit as 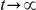 of 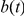 is 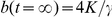. Taking this limit in equations (20) and (21) gives steady state solutions. Furthermore, by setting 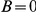, 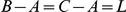, and using dimensionless variables 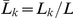, 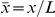, 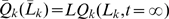, 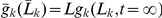 for 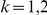 and 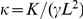, we obtained expressions (2) and (3), displayed earlier in the Results section.

To calculate the animal probability distribution 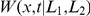, we began by finding the continuous-space limit of the simulation model in the case where the animals and their CPs are infinitely far apart so that they never interact. This corresponds to 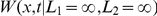, written as 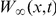 to ease notation.

The master equation for an animal in this limiting case is
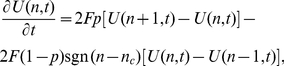
(24)where 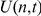 is the probability of the animal being at position 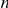 at time 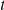, 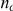 is the position of the CP, 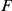 is the jump rate between adjacent lattice sites, and 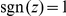 (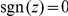, 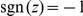) if 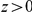 (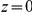, 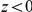). This can be written as
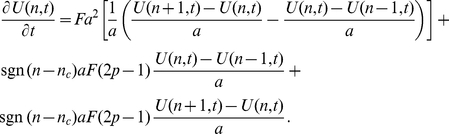
(25)


The continuum limit of (25) can be found by taking the limits as 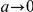, 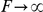, 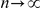, 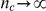 and 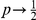 such that 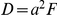, 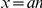, 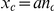 and 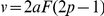 [33], [34]. Physically, 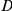 is the diffusion constant of the animal, 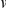 the drift velocity towards the CP, 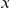 the position of the animal and 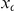 the position of the CP. This procedure leads to the 1D Holgate-Okubo localising tendency model [35], [36]

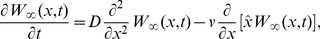
(26)where 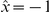, 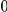 or 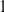 if 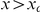, 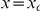 or 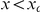 respectively. This has a non-trivial steady state solution [9], proportional to 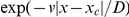. Since the animal is constrained to move between the borders at 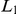 and 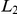, the probability distribution must be zero for 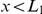 and 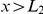. As the solution is a steady state, the flux across 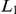 and 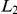 is automatically zero so it suffices to ensure that the integral of the probability distribution between 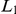 and 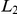 is equal to 1. This leads to the steady state solution 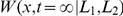 for the Holgate-Okubo localising tendency model within fixed borders
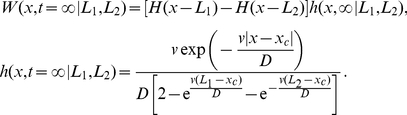
(27)


Using dimensionless variables 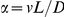, 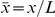, 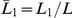, 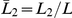, 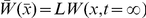, 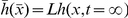 and setting 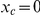 for simplicity, we obtain equation (4) from the results section.

### The reduced analytical model in 2D

In 2D we modelled each territory as a circle with fluctuating radius and the CP at the centre of the circle, assumed to be at the origin for simplicity. As in the 1D scenario, we used an adiabatic approximation so that 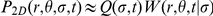, where 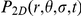 is the joint probability distribution of the animal to be at position 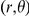 in polar coordinates at time 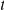 and the territory radius to be 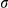. 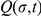 is the probability of the territory radius to be 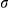 at time 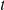 and 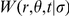 is the probability of the animal to be at position 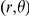 at time 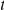 in a territory of fixed radius 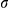.

Similar to the 1D scenario, 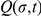 was modelled using a Fokker-Planck formalism with the radius fluctuating around an average value of 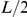, where 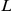 is the distance between adjacent CPs. As such, it can be calculated using the methods of the previous subsection to be
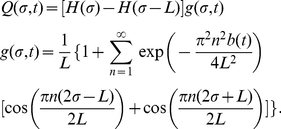
(28)


As the territories are tagged particles in a 2D exclusion process [6], we have 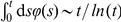 [7]. Taking the limit 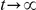 in equation (28) gives a steady state solution. This gives rise to the expression (6) from the main section by using dimensionless variables 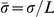, 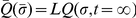, 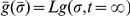.

Following our methods in 1D, we calculated 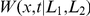 by first taking the continuum limit of the master equation governing the movement of an animal unconstrained by other territories (i.e. 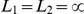). This master equation is
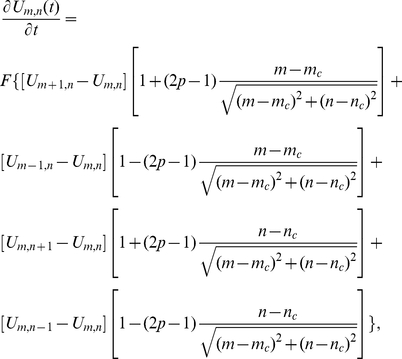
(29)where 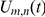 is the probability of the animal being at position 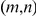 at time 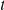 and 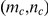 is the position of the CP. To find the continuum limit, this is re-written as follows
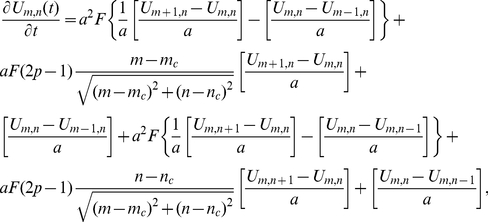
(30)and then the limit as 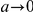, 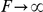, 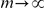, 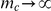, 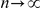, 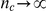 and 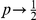 such that 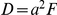, 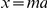, 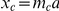, 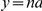, 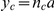 and 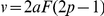 is found. This procedure gives the 2D Holgate-Okubo localising tendency model
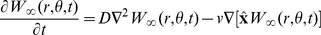
(31)where 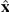 is the unit vector pointing from the animal at 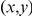 towards the CP at 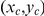, or the zero vector if 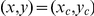, and 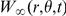 is the probability distribution 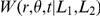 in the limit as 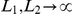 where there is no interaction with other animals. As in 1D, (31) has a non-trivial steady state solution [9], which is proportional to 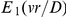. The boundary condition ensuring that 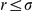, so that the animal is within its territory, is imposed by normalising the steady state solution so that the integral over the circle, of radius 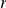 centred at 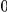, is equal to 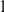. This leads to the following steady state solution 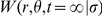 for 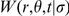


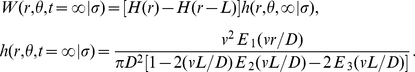
(32)


By using dimensionless variables 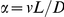, 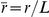, 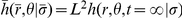, we obtained equation (7) from the results section.
